# Function and molecular mechanism analysis of *Ca*LasSDE460 effector involved in the pathogenesis of “*Candidatus* Liberibacter asiaticus” in citrus

**DOI:** 10.1186/s43897-023-00062-3

**Published:** 2023-07-24

**Authors:** Shuai Wang, Meixia Du, Liting Dong, Rongrong Qu, Danlu Ran, Juanjuan Ma, Xuefeng Wang, Lanzhen Xu, Weimin Li, Yongrui He, Xiuping Zou

**Affiliations:** 1 Integrative Science Center of Germplasm Creation in Western China (CHONGQING) Science City, Citrus Research Institute, Southwest University/National Citrus Engineering Research Center, Chongqing, People’s Republic of China; 2 Key Laboratory for Northern Urban, Agriculture of Ministry of Agriculture and Rural Affairs, Beijing University of Agriculture, Beijing, People’s Republic of China

**Keywords:** Citrus, HLB, *Ca*LasSDE460, Transcriptome, Ectopic expression

## Abstract

Citrus Huanglongbing (HLB), caused by *Candidatus* Liberibacter asiaticus (*Ca*Las), is the most serious disease worldwide. *Ca*LasSDE460 was previously characterized as a potential virulence factor of *Ca*Las. However, the function and mechanism of *Ca*LasSDE460 involved in *Ca*Las against citrus is still elusive. Here, we showed that transgenic expression of *Ca*LasSDE460 in Wanjincheng oranges (*C. sinensis* Osbeck) contributed to the early growth of *Ca*Las and the development of symptoms. When the temperature increased from 25 °C to 32 °C, *Ca*Las growth and symptom development in transgenic plants were slower than those in WT controls. RNA-seq analysis of transgenic plants showed that *Ca*LasSDE460 affected multiple biological processes. At 25 °C, transcription activities of the “Protein processing in endoplasmic reticulum” and “Cyanoamino acid metabolism” pathways increased while transcription activities of many pathways decreased at 32 °C. 124 and 53 genes, separately annotated to plant-pathogen interaction and MAPK signaling pathways, showed decreased expression at 32 °C, compared with these (38 for plant-pathogen interaction and 17 for MAPK signaling) at 25 °C. Several important genes (MAPKKK14, HSP70b, NCED3 and WRKY33), remarkably affected by *Ca*LasSDE460, were identified. Totally, our data suggested that *Ca*LasSDE460 participated in the pathogenesis of *Ca*Las through interfering transcription activities of citrus defense response and this interfering was temperature-dependent.

**Graphical Abstract:**

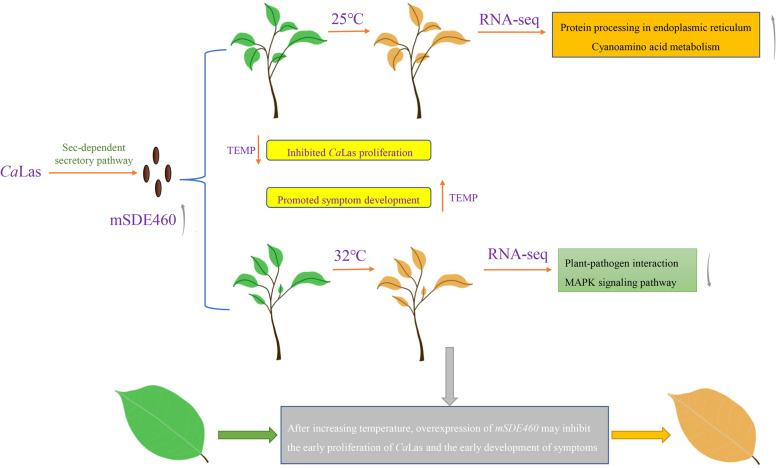

**Supplementary Information:**

The online version contains supplementary material available at 10.1186/s43897-023-00062-3.

## Core

*Ca*LasSDE460 promotes the early growth of *Candidatus* Liberibacter asiaticus and the development of HLB symptoms through interfering transcription activities of plant defense response and this interfering was temperature-dependent in citrus.

## Gene and accession numbers

Sequence data from this article can be found in the database of the National Center for Biotechnology (NCBI) under the accession numbers:

- CaLasSDE460 (CLIBASIA_00460)
- RNA-seq raw data (accession number PRJNA992761).
- A list of genes used in the qRT-PCR analysis can be found in Tables 1 and 2.

**Table 1:** The representative DEGs involved in citrus defense response in OE-5 transgenic plant compared to WT control

Gene Id	Description	log2 (Fold change)	
		25 °C	32 °C
Hormone metabolism			
Cs_ont_5g033020	NCED3, a key enzyme for abscisic acid biosynthesis	-2.00	6.73
Cs_ont_2g025170	GRAM domain family protein	-1.92	1.63
Cs_ont_1g027050	CsSAMT1 methylating salicylic acid	-2.35	-6.98
Cs_ont_2g022650	UDP-Glycosyltransferase affecting auxin homeostasis	1.07	4.83
Cs_ont_2g033670	LOX2 for jasmonic acid accumulation	-1.86	-5.95
Cs_ont_7g001050	HXXXD-type acyl-transferase family protein	1.33	-2.03
Cell wall			
Cs_ont_7g027340	UDP-glucose 6-dehydrogenase family protein	-1.47	2.38
Cs_ont_5g012770	Xyloglucan galactosyltransferase	-2.67	1.21
Cs_ont_5g007250	A protein similar to a beta-xylosidase	2.00	-1.20
Cs_ont_4g026360	Xyloglucan endotransglycosylase-related protein	-1.90	1.34
Cs_ont_3g025070	Beta-d-xylosidase	1.18	-2.67
Redox glutaredoxin			
Cs_ont_2g027370	Regulation of protein redox state	-1.94	2.38
Cs_ont_4g017280	Glutathione transferases	-1.33	3.00
Cs_ont_7g025640	Early-responsive to dehydration 9 (erd9)	-1.34	1.75
Signaling			
Cs_ont_6g012480	Calcium-binding allergen Bet v 3 (Bet v III)	-1.58	-2.22
Cs_ont_3g000240	Calmodulin like 37 (CML37)	-2.15	2.27
Cs_ont_4g012420	Calcium-binding EF-hand family protein	-2.56	1.34
Cs_ont_3g011780	A Rho GTPase-activating protein	-2.41	1.55
MAPK			
Cs_ont_8g000450	Mitogen-activated protein kinase kinase kinase 14 (MAPKKK14)	-1.69	4.70
Defense gene			
Cs_ont_5g034570	Kunitz family trypsin and protease inhibitor protein	1.86	-2.40
Heat shock protein			
Cs_ont_7g003880	Heat shock cognate protein 70–1 (HSC70-1)	-2.82	1.26
Cs_ont_1g010380	Heat shock protein 70 (HSP70b)	1.51	4.50

**Table 2:** The representative DEGs involved in citrus defense response in WT and OE-5 transgenic plant at 32 °C

Gene Id	Description	log2 (Fold change)	
		WT	OE-5
Hormone metabolism			
Cs_ont_5g033020	NCED3, a key enzyme for abscisic acidbiosynthesis	-4.11	6.73
Cs**_**ont**_**2g025170	GRAM domain family protein	-2.15	1.49
Cs_ont_1g027050	CsSAMT1 methylatingsalicylic acid	1.33	-2.49
Cs_ont_2g022650	UDP-Glycosyltransferase affecting auxin homeostasis	1.93	5.49
Cell wall			
Cs**_**ont**_**7g027340	UDP-glucose 6-dehydrogenase family protein	-1.79	2.11
Cs_ont_3g025070	Beta-d-xylosidase	1.74	-3.49
Cs**_**ont**_**4g026430	Hydrolase activity	7.04	-1.86
Redox glutaredoxin			
Cs**_**ont**_**2g027370	Regulation of protein redox state	-1.06	3.57
Cs_ont_4g017280	Glutathione transferases	-1.25	3.09
Signaling			
Cs**_**ont**_**6g012480	Calcium-binding allergen Bet v 3 (Bet v III)	-2.54	-3.21
Cs_ont_3g000240	Calmodulin like 37 (CML37)	-2.61	2.00
Cs**_**ont**_**4g012420	Calcium-binding EF-hand family protein	-2.18	1.72
Protein degradation			
Cs**_**ont**_**7g024750	Ubiquitin-protein ligase activity	-4.54	3.89
Misc.functions			
Cs**_**ont**_**6g018900	Glutathione transferase L3 (GSTL3)	-1.35	-8.75
Abiotic stress			
Cs**_**ont**_**5g008470	Encodes a membrane-bound protein	-1.43	6.27

## Introduction

Citrus Huanglongbing (HLB) disease was first discovered in southern China in 1919 and spread rapidly in Asia (Jones et al., 2021). So far, the HLB disease has been found in Africa, America and other regions and has caused devastating damage on the citrus industry all over the world (Hong et al., 2019). HLB pathogen is a non-culturable phloem-limited *Candidatus* Liberibacter bacterium, which includes Asian species (“*Candidatus* Liberibacter asiaticus”, *Ca*Las), African species (*Candidatus* Liberibacter africanus, *Ca*Laf) and American species (*Candidatus* Liberibacter americanus, *Ca*Lam) (Bové et al., 2006). *Ca*Las, which can infect all citrus cultivars, is the most widely distributed and harmful in citrus. The bacterium is widely transmitted by the phloem feeding psyllid *Diaphorina citri Kuwayama* in field (Bové et al., 2006). Infecting plants usually displayed yellow shoots, blotchy mottle leaves, greening and lopsided fruits, aborted seeds, decayed roots and premature death (Bové et al., 2006; da et al., 2016). Still now, *Ca*Las can not be cultured in vitro, which seriously hinders the research on the pathogenic mechanism and HLB disease resistance mechanism in citrus. It is very difficult for chemical bactericides to enter the plant to effectively kill pathogens because *Ca*Las lived in the phloem of citrus (Riera et al. 2017). There is no cures or resistant cultivars available for citrus farmers.

The genomes of many *Ca*Las strains were published since Duan et al. (2009) first successfully sequenced the whole genome of one *Ca*Las strain using metagenomics method (Pitino et al. 2016, 2018; Prasad et al. 2016). These data showed that all the strains contained genes encoding the complete Sec-dependent secretion system. The system can transfer Sec-dependent effectors (SDEs) including virulence factors into plant cells to participate in pathogen infection, growth and spread (Costa et al. 2015; Green et al., 2016). Among other phloem-limited pathogens such as phytoplasma, some SDEs have been shown to be critical for pathogenicity (Hogenhout et al., 2008). By combining bioinformatics prediction with experiments based on *E. coli* alkaline phosphatase (PhoA) fusion, 86 putative SDEs proteins were discovered in *Ca*Las and had functional Sec-dependent secretion signal peptides (Prasad et al. 2016). Increasing studies demonstrate that SDEs play an important role in the pathogenesis of *Ca*Las in citrus (Pagliaccia et al. 2017). CLIBASIA_05315 effector induced starch accumulation, cholorosis and cell death in tobacco (Pitino et al. 2016; Pitino et al. 2018). This effector was later shown to associate papain-like cysteine proteases and regulates host defense response in citrus (Clark et al. 2018). LasP235 effector from *Ca*Las prophage maybe involved in regulation of HLB symptom development through targeting several citrus innate immune proteins (Hao et al., 2019). CLIBASIA_05115 positively regulated early pathogenic colonization of citrus by modulating the transcriptional regulation of genes involved in SAR responses (Du et al., 2022). CLIBASIA_03135 (lotP) mediates citrus defense response by interacting with several plant chaperons displaying proteolytic activities (Loto et al. 2017).

It was shown that *Ca*LasSDE460 (CLIBASIA_00460) is a Sec-dependent secretory protein (Shi et al. 2019). Recently, Liu et al. (2019) showed that *mSDE460*, the mature secretion protein of *Ca*LasSDE460, can cause chlorosis and necrosis in tobacco leaves and suggested that the accumulation of *mSDE460* in the nucleus was positively correlated with its pathogenicity in plants. However, the function and mechanism of action of *Ca*LasSDE460 in *Ca*Las infection in citrus are still unclear. And the relationship between the accumulation of *mSDE460* in the nucleus and its pathogenicity in citrus is also unknown.

In this study, thus, we investigated the potential role of *Ca*LasSDE460 in interaction of *Ca*Las and citrus through ectopic expression of *mSDE460* in HLB-susceptible Wanjincheng oranges (*C. sinensis* Osbeck). Our data showed that the ectopic expression of *mSDE460* favored pathogen proliferation and symptoms development in transgenic plants infected by *Ca*Las. And after increasing temperature, ectopic expression of *mSDE460* may inhibit the early proliferation of *Ca*Las and early development of HLB symptoms. Molecular mechanism of *Ca*LasSDE460 pathogenicity was further determined by RNA-seq analysis of transgenic plants.

## Results

### Generation of transgenic citrus overexpressing *mSDE460*

**Fig. 1:**
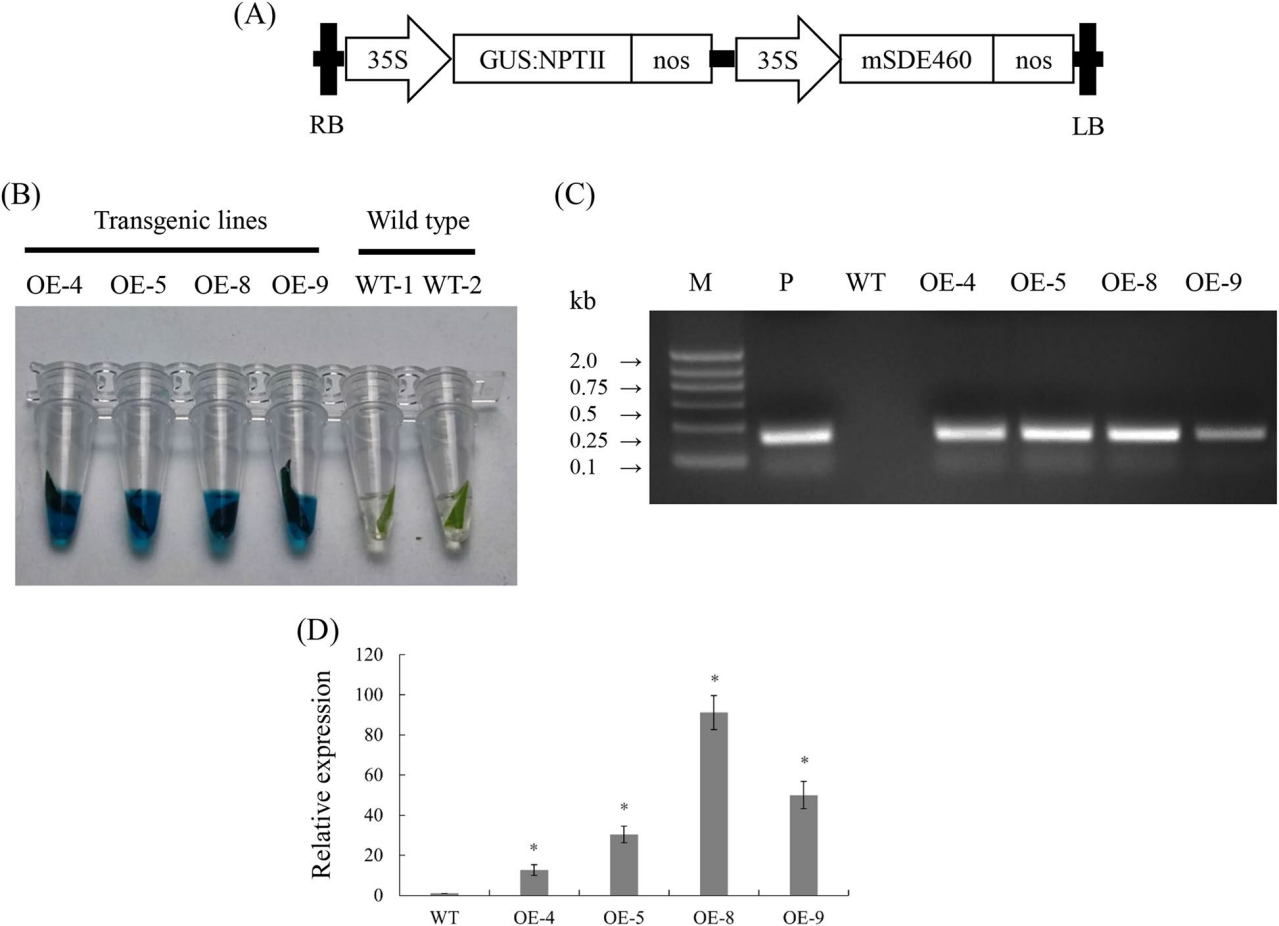
Wanjincheng orange transgenic plants overexpressing *mSDE460*. **A** T-DNA structure of plant expression vector for the genetic transformation of citrus. A 35S, CaMV 35S promoter; GUS:NPTII, fusion of β-glucuronidase and neomycin phosphotransferase genes (for the screening of citrus transformants); *mSDE460*, the coding sequence of *Ca*LasSDE460 mature protein; NOS, the nopaline synthase terminator; LB, left border; RB, right border. **B** Screening of transgenic plants by GUS histochemical staining. The blue stains indicated transformants. **C** Identification of transgenic plants by PCR. M, DNA marker, P, *p35S:mSDE460* plasmid; WT, wildtype control; OE-#, transgenic plants. **D** Relative expression levels of *mSDE460* in transgenic plants. Relative expression of *Ca*LasSDE460 in transgenic plans was normalized against its expression in the WT using the citrus GAPDH gene (Mafra et al., 2012) as internal reference. Bars represent the average ± standard error of the means(*n* = 3). The primers used in (**C**) and (**D**) were listed in Supplementary Table 1. The asterisks indicate significant differences compared to WT control (*p* < 0.05, Student’s *t*-test)

**Fig. 2:**
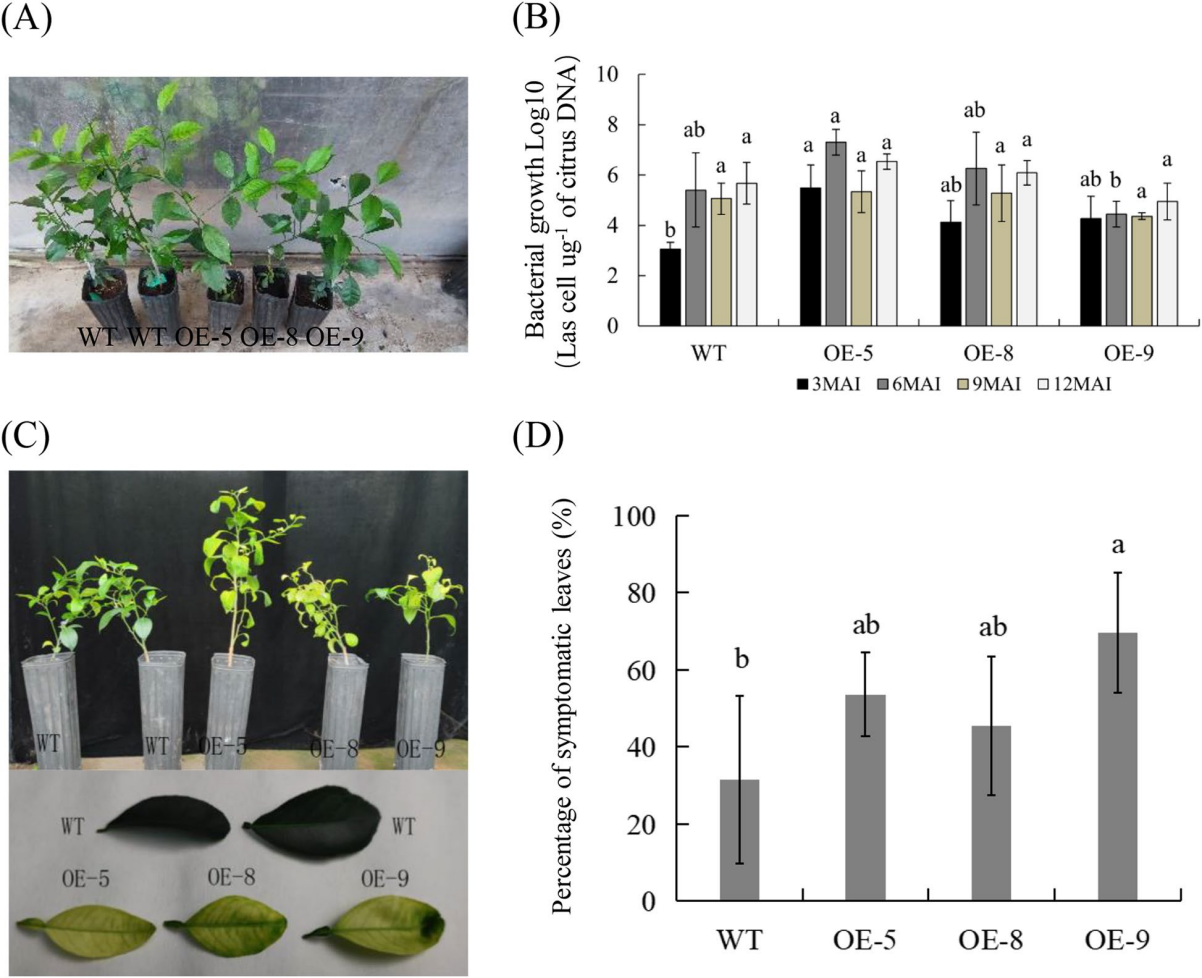
Evaluation of Citrus HLB-resistance in transgenic plants. **A** Healthy wild-type (WT) and transgenic plants before *Ca*Las-infected. **B** Quantitative analysis of *Ca*Las growth in transgenic plants at 3, 6, 9 and 12 months after infection (MAI). The bacterial populations (*Ca*Las cells µg.^−1^ of citrus DNA) were determined using qPCR. **C** HLB symptoms in the transgenic plants and WT controls at 9 MAI. **D** Statistic analysis of symptoms in the leaves at 16 MAI. Percentage (%) of symptomatic leaves per plant was calculated by the number of symptomatic leaves out of the total number of leaves. Bars represent the average ± standard error of the means from three plants per line (*n* = 3). WT, wild type; OE-#, transgenic plants. Different letters at the top of the bars indicate significant differences from the WT control (*p* < 0.05, Student’s *t*-test)

To understand the functions of *Ca*LasSDE460 in citrus, the *mSDE460* gene, encoding the mature protein *Ca*LasSDE460, was controlled by a strong promoter 35S (Fig. 1A), and was introduced into HLB-susceptible Wanjincheng oranges via *Agrobacterium tumefaciens*-mediated transformation. In this study, four transgenic lines (OE-4, OE-5, OE-8 and OE-9) were identified by GUS histochemical staining and PCR amplification (Fig. 1B and C). Expression levels of *mSDE460* in these transgenic lines were verified by RT-qPCR. The OE-4, OE-5, OE-8, and OE-9 lines had significantly higher transcriptional levels of *mSDE460*, compared with the WT control (Fig. 1D). Here, OE-5, OE-8 and OE-9 lines were investigated in the following experiments. No obvious phenotypical differences were detected in transgenic plants compared with WT ones in greenhouse (Fig. 2A).

### Ectopic expression of *mSDE460* enhanced symptom development in transgenic plants infected by *Ca*Las

To evaluate the role of *Ca*LasSDE460 on *Ca*Las growth and symptom development, the OE-5, OE-8, and OE-9 transgenic lines (Fig. 2A) were infected by *Ca*Las using the inoculation method (Cifuentes-Arenas et al. 2019). At 3, 6, 9 and 12 months after infection, *Ca*Las growth in transgenic plants were determined by qPCR. At three months after infection, the *Ca*Las contents in transgenic plants was higher than that in wild-type plants, and OE-5 transgenic plants had significantly increased *Ca*Las contents compared to WT plants (Fig. 2B). However, there was no significant difference in the pathogen content between transgenic plants and wild type plants from 6 to 12 months after *Ca*Las infection.

Six months after infection, HLB symptoms (such as chlorosis or mottled yellow leaves) began to appear in some leaves of *mSDE460* transgenic plants, while no obvious HLB symptoms were observed in the WT control leaves. After nine months of infection, most of leaves and new flush from transgenic plants demonstrated HLB symptoms, which were more serious than that in WT controls (Fig. 2B). Among 16 months of greenhouse evaluation, transgenic plants continuously displayed serious symptoms compared to WT controls. At 16 months of infection, we counted the number of leaves with HLB-symptoms in transgenic plants. The results showed that 53.62%, 45.49% and 69.62% leaves from the OE-5, OE-8 and OE-9 lines had symptoms, which were more than that (31.43%) of WT controls (Fig. 2C). These data indicated that *Ca*LasSDE460 was involved in the development of HLB symptoms.

### Effects of temperature on the function of *Ca*LasSDE460 effector in citrus

**Fig. 3:**
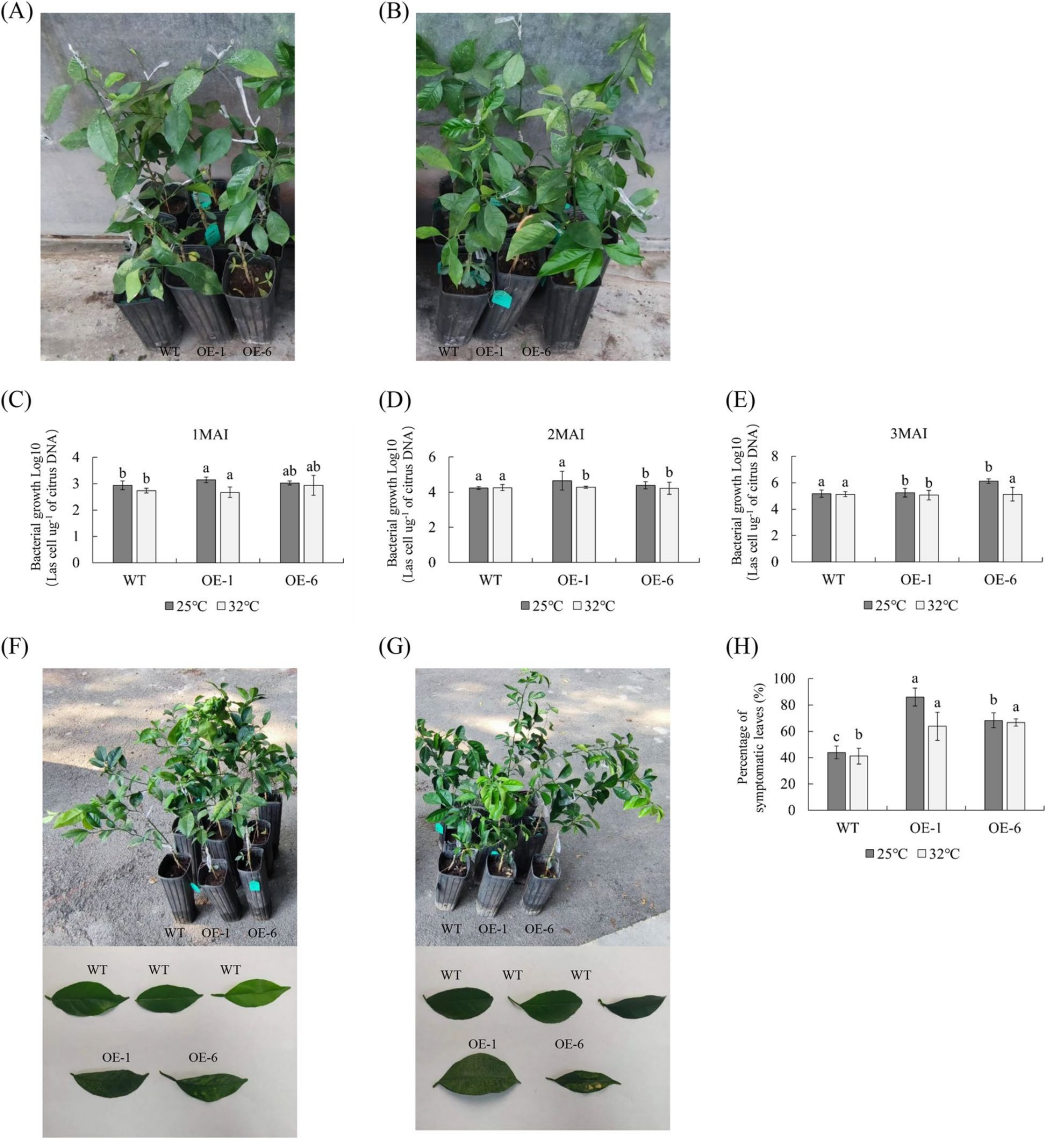
Evaluation of Citrus Huanglongbing (HLB) resistance in *Ca*LasSDE460 transgenic plants under 25 °C and 32 °C treatment for three months. **A** and **B** Healthy wild-type (WT) and transgenic plants before *Ca*Las-infected. **C** Quantitative analysis of *Ca*Las contents in WT and transgenic plants under 25 °C and 32 °C treatment for one month. **D** Quantitative analysis of *Ca*Las contents in WT and transgenic plants under 25 °C and 32 °C treatment for two months. **E** Quantitative analysis of *Ca*Las contents in WT and transgenic plants under 25 °C and 32 °C treatment for three months. **F** HLB symptoms in the transgenic plants and WT controls under 25 °C treatment for three months. **G** HLB symptoms in the transgenic plants and WT controls under 32 °C treatment for three months. The bacterial populations (*Ca*Las cells µg.^−1^ of citrus DNA) were determined using qPCR. (H) Statistic analysis of symptoms in the leaves under 25 °C and 32 °C treatment for three months. Percentage (%) of symptomatic leaves per plant was calculated by the number of symptomatic leaves out of the total number of leaves. Bars represent the average ± standard error of the means from three plants per lines(*n* = 3). WT, wild type; OE-#, transgenic plants. Different letters at the top of the bars indicates significant differences from the WT control (*p* < 0.05, Student’s* t*-test)

To evaluate the effect of temperature on *Ca*Las growth in transgenic plants, three plants of each transgenic line (OE-1 and OE-6) and WT control were inoculated with *Ca*Las and were grown at 25 °C (A) and 32 °C (B) for 3 months. From 1 to 3 month after infection, the pathogen contents increased gradually in transgenic and WT plants (Fig. 3C-E). However, the pathogen contents in transgenic plants was higher than that in wild-type plants at 25 °C (Fig. 3C-E). This indicated that *mSDE460* expression promoted *Ca*Las proliferation. When the temperature increased from 25 °C to 32 °C, *Ca*Las proliferation was slower in transgenic plants than in WT plants (Fig. 3C-E).

Three months after infection, some leaves of *mSDE460* transgenic plants at 25 °C and 32 °C began to appear obvious HLB symptoms, while no obvious HLB symptoms were observed in the wild-type control leaves (Fig. 3F and G). We counted the number of leaves with HLB symptoms in transgenic plants. At 25 °C, 80.91% and 74.19% of the leaves from the OE-1 and OE-6 lines showed symptoms, which were remarkedly more than that (43.97%) of WT plants. At 32 °C, 76.19% and 67.61% of the leaves from the OE-1 and OE-6 lines showed symptoms, which were still more than that (41.19%) of WT plants (Fig. 3H). These data indicated that HLB symptoms development was faster in transgenic plants than that in WT plants.

Overall, these data domonstrated that high temperature (32 °C) inhibited the function of *Ca*LasSDE460 effector in promoting *Ca*Las pathogenicity in citrus plants.

### An overview of transcriptomic changes in transgenic plants

**Fig. 4:**
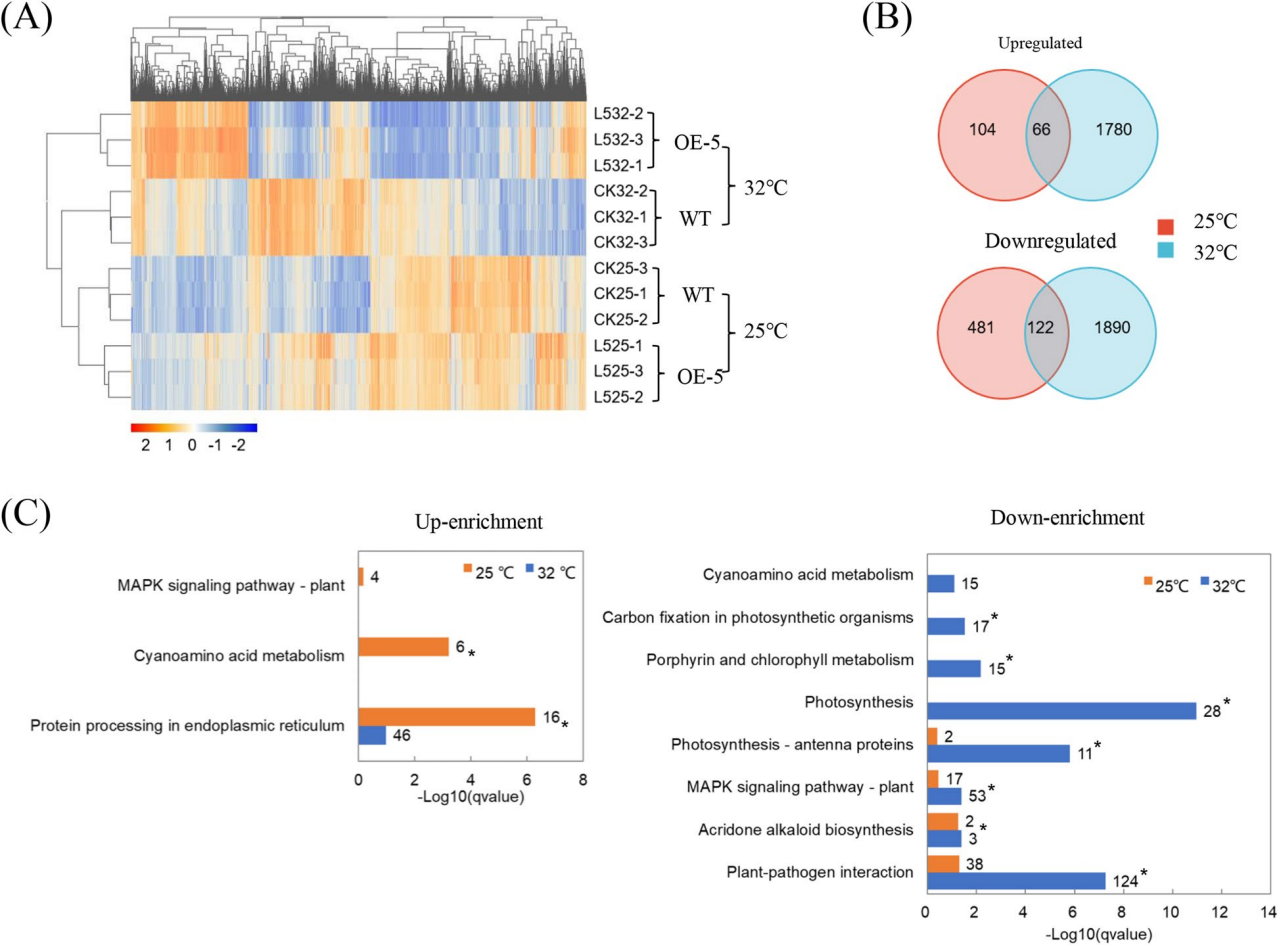
The overall gene expression profile of transgenic citrus overexpressing *Ca*LasSDE460 compared to wild-type (WT) control. **A** Heatmap analysis of differentially expressed genes (DEGs) between transgenic lines and WT lines at 25 °C and 32 °C. Transgenic lines showed a similar hierarchical clustering pattern. **B** Venn diagrams showed the overlaps of DEGs between 25 °C and 32 °C treatment. 66 upregulated and 122 downregulated DEGs were identified at both 25 °C and 32 °C treatment. **C** Enrichment comparison of the KEGG pathways between 25 °C and 32 °C treatment in the transgenic plant. All the DEGs were used to the analysis. The representative KEGG pathway having up-regulated (Up-enrichment) and down-regulated (Down-enrichment) were presented here. *indicates differentially represented KEGG pathways (q-value < 0.05, Fisher’s exact test)

To understand molecular mechanism of *Ca*LasSDE460 involved in *Ca*Las pathogenicity, we performed RNA-seq using the OE-5 line and WT control. Liu et al. (2019) suggested that nuclear localization of *mSDE460* protein was temperature-dependent and positively correlated with its pathogenicity in tobacco. Here, we also compared transcriptomic differences of the OE-5 line at 25 °C and 32 °C treatments. Totally, about 20 million clean reads were obtained in each sample (Supplementary Fig. 1 and Supplementary Tables 2 and 3). Cluster Heatmap analysis showed that transcriptomic profiling of transgenic plant was significantly different compared with WT at either 25 °C or 32 °C and they also displayed significant changes between 25 °C and 32 °C treatments (Fig. 4A). At 25 °C, a total of 773 differentially expressed genes (DEGs) were identified in the transgenic plant, of which 170 DEGs were up-regulated and 603 DEGs were down-regulated when compared with WT control (Fig. 4B and Supplementary Table 4). When the temperature increased to 32 °C, the number of DEGs markedly increased in the transgenic plant and 3858 DEGs were scored. At this condition, 1846 DEGs were up-regulated and 2012 DEGs were down-regulated compared with WT control (Fig. 4B and Supplementary Table 5). In the transgenic plant, 122 up-regulated and 66 down-regulated DEGs were shared by 25 °C and 32 °C treatments.

KEGG pathway enrichment analysis of DEGs showed that many pathways were significantly affected by *mSDE460* expression. Cyanoamino acid metabolism, Amino sugar and nucleotide sugar metabolism, Monoterpenoid biosynthesis and Plant hormone signal transduction pathways were significantly enriched in transgenic plants at 25 °C while Photosynthesis, Photosynthesis-antenna proteins, Porphyrin and chlorophyll metabolism, Plant-pathogen interaction and Plant hormone signal transduction pathways were significantly enriched at 32 °C treatments (Supplementary Fig. 2 and Supplementary Table 6). The data showed that Plant hormone signal transduction pathway was significantly regulated by *mSDE460* expression either at 25 °C or 32 °C treatment. At 25 °C treatment, 16 and 6 up-regulated DEGs were significantly enriched in “protein processing in endoplasmic reticulum” and “Cyanoamino acid metabolism” pathways of the transgenic plant, respectively, which were not identified at 32 °C treatment (Fig. 4C). We further discovered that many down-regulated DEGs were significantly enriched in many pathways at 32 °C, compared to 25 °C treatment (Fig. 4C). 17, 15, 28 and 11 down-regulated DEGs were significantly enriched in Carbon fixation in photosynthetic organisms, Porphyrin and chlorophyll metabolism, Photosynthesis and Photosynthesis-antenna proteins, respectively, indicating that expression of *mSDE460* suppressed photosynthesis-related process of transgenic plants at 32 °C. Especially, at 32 °C, 124 and 53 down-regulated DEGs were significantly enriched in Plant-pathogen interaction and MAPK signaling pathway-plant pathways, respectively (Fig. 4C).

**Fig. 5:**
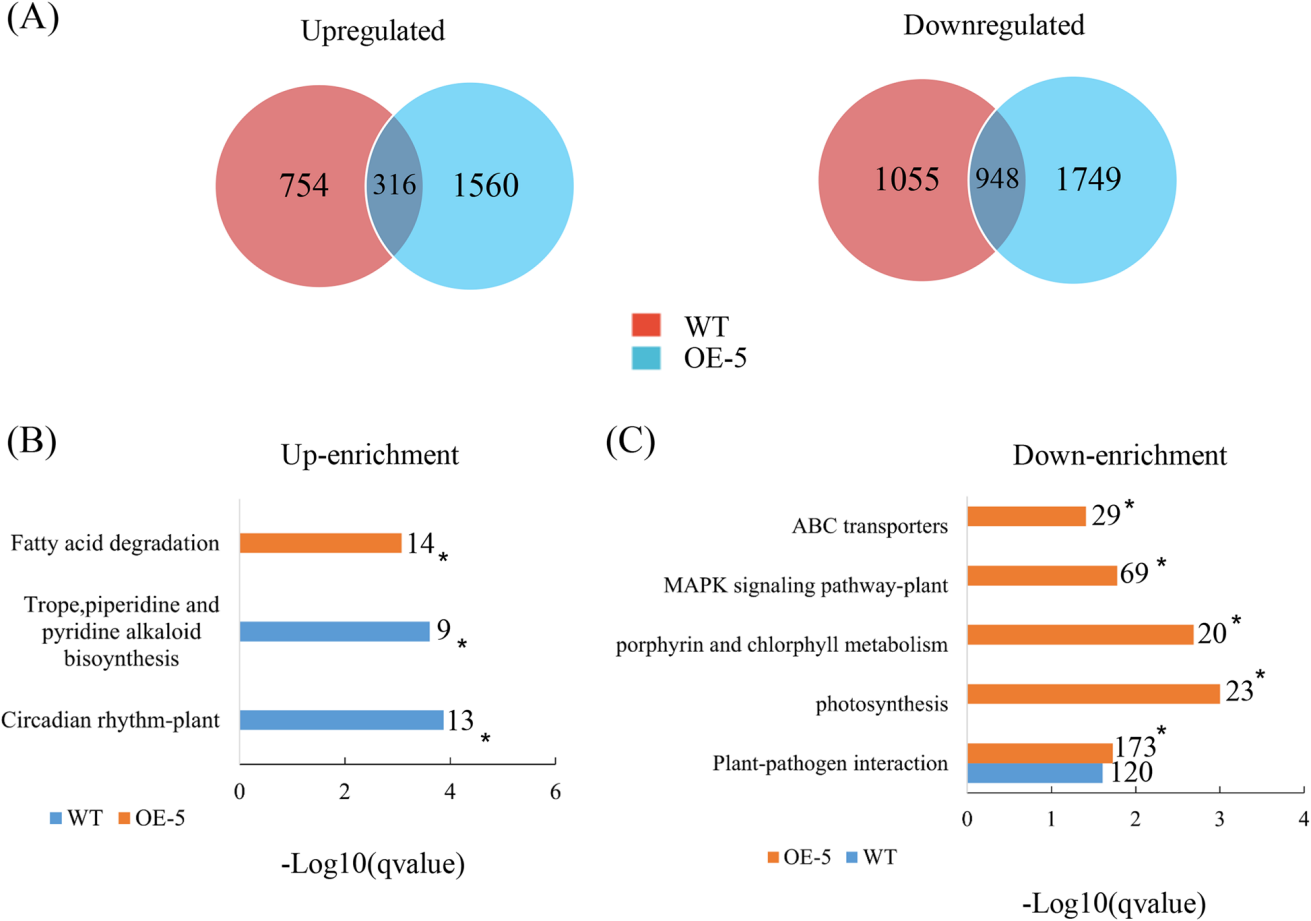
Transcriptomic characteristics of transgenic citrus overexpressing *Ca*LasSDE460 in response to temperature compared to wild-type (WT) control. **A** Venn diagrams showing the overlaps of differentially expressed genes (DEGs) between WT and OE-5 transgenic plants at 32 °C compared to 25 °C. 316 upregulated and 948 downregulated DEGs were identified at WT and OE-5 transgenic plants, respectively. **B** and **C** enrichment comparison of the KEGG pathways between WT and OE-5 transgenic plants. All the DEGs were used to the analysis. The representative KEGG pathways having up-regulated (Up-enrichment) and down-regulated (Down-enrichment) were presented here. * indicates differentially represented KEGG pathways (q-value < 0.05, Fisher’s exact test)

To characterize transcriptional activities affected by *Ca*LasSDE460 in response to temperature, we further compared the transcriptomic changes between WT and OE-5 transgenic plant at 32 °C, using those at 25 °C as control. In total, 3073 and 3573 DEGs were identified in WT and OE-5 plant, respectively (Fig. 5A and Supplementary Table 7 and 8). In both WT and OE-5 plant, the number of down-regulated DEGs was more than that of up-regulated ones at 32 °C (Fig. 5A). KEGG analysis of DEGs showed that the Plant-pathogen interaction pathway was significantly enriched in both WT and OE-5 plants while Porphyrin and chlorophyll metabolism, starch and sucrose metabolism, photosynthesis, thiamine metabolism, folate biosynthesis, fatty acid degaradation, Cyanoamino acid metabolism and Fatty acid elongation pathways were significantly enriched in OE-5 plant (Supplementary Fig. 3). In OE-5 transgenic plant, up-regulated DEGs were only significantly enriched in the fatty acid degradation pathway, and in WT plant, up-regulated DEGs were significantly enriched in Trope, piperidine and pyridine alkaloid biosynthesis pathways (Fig. 5B and Supplementary Table 9). However, in OE-5 plant, down-regulated DEGs were significantly enriched in ABC transporters, MAPK signaling pathway-plant, Porphyrin and chlorophyll metabolism, photosynthesis, and plant-pathogen interaction pathways while no down-regulated DEGs were significantly enriched in these pathways in WT plant (Fig. 5C and Supplementary Table 9). These data indicated that *mSDE460* overexpression mainly negatively transcription activities in plant-pathogen interaction and MAPK signaling pathway-plant pathways, which was high temperature-dependent.

### Transcriptional characteristics of defense response in transgenic plants

**Fig. 6:**
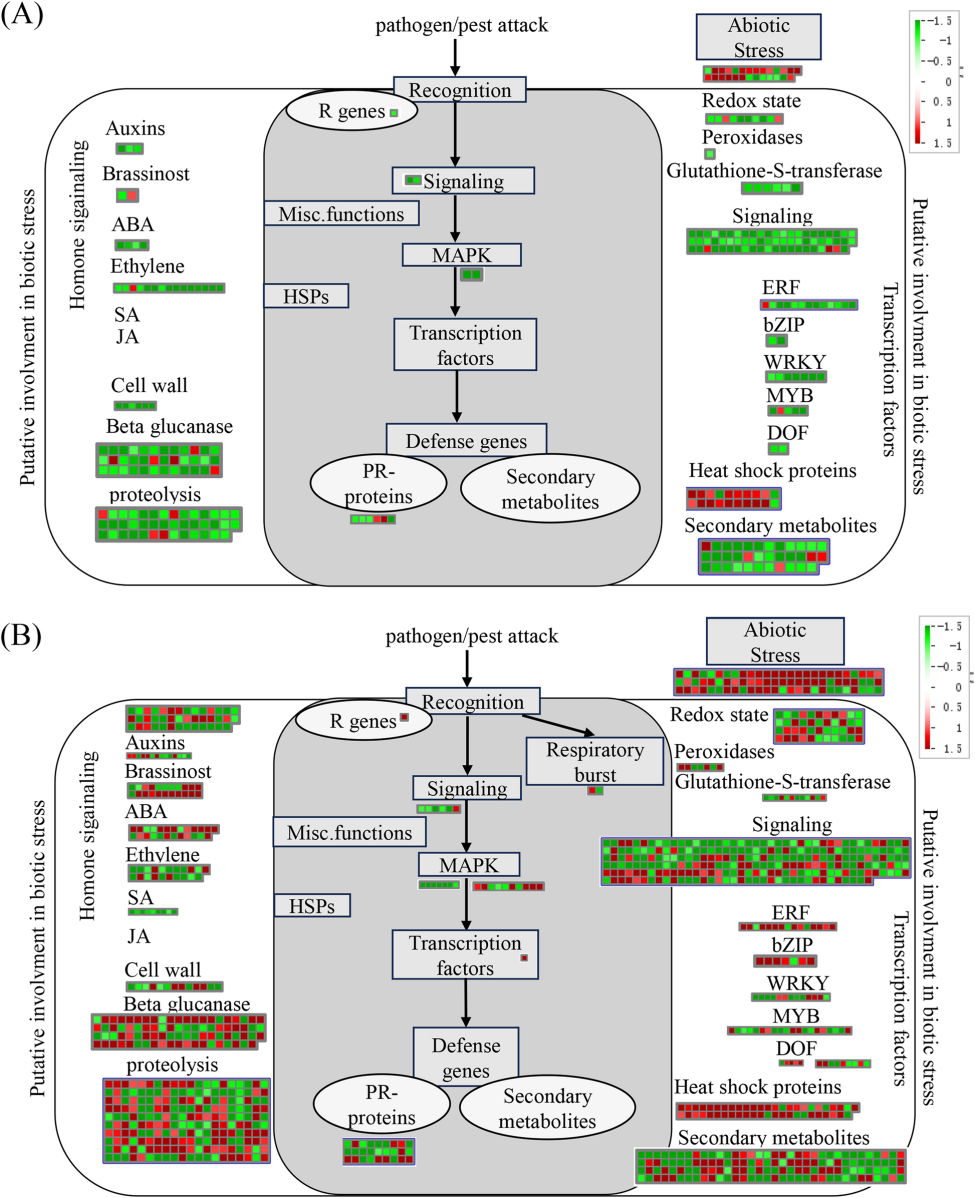
MapMan visualizes the functional categories of differentially expressed genes in transgenic citrus with expression of *Ca*LasSDE460 at 25 °C (**A**) and 32 °C (**B**), compared to wild-type plants. Every square block indicates a DEG. Significantly up-regulated and down-regulated genes are displayed in red and green, respectively

These DEGs, which were related to biological stress, were further functionally classified using MapMan software (Fig. 6 and Supplementary Fig. 4). No obvious difference in transcriptional activities of defense response were detected between WT and transgenic plant OE-5 when temperature increased from 25 °C to 32 °C (Fig. 6 and Supplementary Fig. 4). When using WT plant as control, at 25 °C, 318 DEGs were assigned to biological stress process, of which 66 DEGs were up-regulated and 252 DEGs were down-regulated, indicating *mSDE460* expression mainly negatively regulated biological stress-related transcriptional activities (Fig. 6A). When the temperature was 32 °C, 756 DEGs were assigned to biological stress process, of which 328 genes were up-regulated and 428 genes are down-regulated (Fig. 6B). At 25 °C, only nine DGEs (1 R gene, 2 signaling genes and 6 PR-proteins genes) were assigned to “Pathogen/pest attack” biological process, which directly participated in plant defense response (Fig. 6A). But at 32 °C, 43 DGEs (1 R gene, 6 signaling genes and 27 PR-proteins genes) were detected in this biological process (Fig. 6B). Additionally, many genes in other functional groups, which were related to biological stress, were significantly affected by *mSDE460* expression when the temperature increased from 25 °C to 32 °C (Fig. 6B).

Tables 1 and 2 listed representative DGEs involved in citrus defense response. The genes showed significantly different expressions at both 25 °C and 32 °C, and assigned to hormone metabolism, cell wall, redox glutaredoxin, signaling, MAPK signaling pathway, defense gene and heat shock protein group. When the temperature increased from 25 °C to 32 °C, 17 DGEs were significantly up-regulated by more than 3 times, and 4 DGEs were significantly down-regulated by more than 3 times. *Cs_ont_5g033020* encoding a key enzyme of abscisic acid biosynthesis was down-regulated by 2.00-fold at 25 °C and when the temperature rose to 32 °C, it was up-regulated by 6.73-fold. *Cs_ont_1g027050* and *Cs_ont_2g033670* genes were assigned to salicylic acid (SA) and jasmonic acid (JA) metabolism, respectively, and had decreased expression at 25 °C. But their expressions further decreased at 32 °C. In redox glutaredoxins group, all the three genes were down-regulated at 25 °C, but at 32 °C, their expressions were up-regulated. In signaling, Three DEGs involved in Ca^2+^ signaling showed remarkably different expression between 25 °C and 32 °C treatments. A DEGs (*Cs_ont_8g000450*) encoding a mitogen-activated protein kinase kinase kinase 14 (MAPKKK14), which was assigned to MAPK signaling pathway, was down-regulated by 1.69-fold at 25 °C and when the temperature rose to 32°C, it was up-regulated by 4.69-fold. In defense genes group, the expression of *Cs_ont_5g034570*, belonging to Kunitz family protease inhibitors, increased and decreased at 25 °C and 32 °C, respectively. In heat shock proteins group, the expression level of *Cs_ont_1g010380* encoding a heat shock protein HSP70b increased from 1.51 to 4.50 folds when temperature rose from 25 °C and 32 °C. *Cs_ont_7g003880* encoding Heat shock cognate protein 70–1 (HSC70-1) was down-regulated by 2.86-fold at 25 °C but at 32 °C, it was up-regulated by 1.26-fold. The two genes were assigned to the “protein processing in endoplasmic reticulum” pathway (Supplementary Table 6).

In Table 2, there were significant differences in the expression of DEGs in both WT plant and transgenic plant, including hormone metabolism, cell wall, redox glutaredoxin, signaling, protein degradation, misc.functions and abiotic stress. 10 DGEs in transgenic plant were significantly up-regulated by more than three times compared to WT plant, while 4 DGEs were significantly down-regulated by more than three times. *Cs_ont_7g024750* with ubiquitin ligase activity was down-regulated by 4.54-fold in WT plant and up-regulated by 3.89-fold in transgenic plant. *Cs_ont_6g018900* gene belongs to misc.functions and its expression decreased in WT plant, but its expression further decreased in transgenic plant. In terms of Abiotic stress, *Cs_ont_5g008470*, which encodes membrane binding protein, showed significantly different expression in WT plant and transgenic plant (Supplementary Tables 7 and 8).

The expression levels of these above genes were further identified using RT-qPCR (Supplementary Fig. 5 and 6).

## Discussion

In this study, we displayed that *Ca*LasSDE460 contributed greening, chlorosis or mottled yellow symptoms development in citrus infected by *Ca*Las. Similarly, *Ca*LasSDE460 also induced foliar chlorosis and necrosis in *N. benthamiana* (Liu et al. 2019). Shi et al. (2019) discovered that *Ca*LasSDE460 was highly expressed at early stage of *Ca*Las infection in multiple citrus varieties, including HLB-susceptible Duncan grapefruit and Washington navel orange, tolerant citron and Cleopatra mandarin, and resistant Pomeroy trifoliate and Carrizo citrange. Meanwhile, our data showed that *mSDE460* expression triggered early pathogen proliferation in transgenic plants. Moreover, *Ca*LasSDE460 exists in all *Ca*Las strains reported to date but is absent from *Ca*Lam and *Ca*Laf (Liu et al. 2019). These results indicated that *Ca*LasSDE460 played a positive role in *Ca*Las early colonization and pathogenicity in citrus. “*Candidatus* Liberibacter solanacearum” is another phloem-limited bacterium, which harms the families Solanaceous and Apiaceous crops in different parts of the world (Sumner-Kalkun et al., 2020). A mSDE460 putative homolog, Lso-HPE1 effector from “*Candidatus* Liberibacter solanacearum” were able to repress plant immune response (Levy et al. 2020), indicating that this homolog effectors have conserved pathogenic function as shown in our study.

Some environment factors such as temperature, drought and pathogen stress can change redistribution of proteins in cells (Fujino et al. 2011; Park et al., 2012). For example, A bHLH transcription activator regulates defense response to *Magnaporthe oryzae* by nucleo-cytosolic trafficking in rice (Meng et al. 2020). Liu et al. (2019) indicated that high temperature (32 °C) restricted accumulation of *mSDE460* in the nuclei of tobacco and reducing its nuclear accumulation enhanced its pathogenicity in *N. Benthamiana*. Heat treatment reduced the HLB-associated symptoms and pathogen titer in *Ca*Las-infected citrus plants (Ding et al. 2018; Doud et al. 2017; Hoffman et al. 2013). In this study, we reavealed that increasing the temperature from 25 °C to 32 °C decreased *Ca*Las growth in transgenic plants*.* The above data revealed potential molecular mechanisms of high temperature in controlling citrus HLB through interfering nucleus localization of the *Ca*Las-secreted effectors in host cells. Additionally, mSDE460-mediated suppression of resistance in OE-5 was more significant than that in the other two lines (Fig. 2), although the expression of *mSDE460* was lower in OE-5 compared with the other two lines (Fig. 1). Proteins determine the biological function of genes. A transgene with high transcription level may not necessarily have high protein content, due to transgenic copy number, stability and integrity of transgenic mRNA, and its translation levels (Roichman et al. 2021). Thus, the strong suppression of resistance in OE-5 line maybe because that the line had high level of mSDE460 protein, which is required to be confirmed by western blot analysis in future.

Studies have shown that plant pathogens deliver effectors to subvert host innate immune responses through reprograming host cellular processes or signaling pathways, thereby facilitating pathogen colonize and spread in the host (Hann et al. 2010; Ceulemans et al. 2021; Toruño et al. 2016). Here, we characterized cellular processes or signaling pathways affected by *mSDE460* expression in transgenic citrus plants using RNA-seq. Based on our investigation, *Ca*LasSDE460 effector remarkably affected the plant hormone signal transduction, MAPK signaling, Plant-pathogen interaction and protein processing in endoplasmic reticulum pathways, which played pivotal roles in regulation of plant defense.

When temperature rose 25 °C to 32 °C, MAPK signaling pathway was negatively affected by *mSDE460* expression. It is shown that high temperature promoted nuclear export of *mSDE460* to reduce its pathogenicity (Liu et al. 2019), and also decreased *Ca*Las proliferation in transgenic plants overexpressing *mSDE460*. Thus, it should be in citrus cytoplasm, not nucleus, where *mSDE460* effector disturbed MAPK signaling pathway to regulate *Ca*Las pathogenicity. Further, we identified a DEG (*Cs_ont_8g000450*) encoding MAPKKK14 which was involved in MAPK signaling pathway. Interestingly, its relative expression remarkably increased from -1.69-fold to 4.70-fold when temperature rose from 25 °C to 32 °C. High (32 °C) temperature inhibited *Ca*LasSDE460 effector entry to plant nucleus (Liu et al. 2019). Thus, these results indicates that *mSDE460* effector maybe negatively affected MAPKKK14 expression. In *Arabidopsis*, wounding and insect feeding activates of the MKK3-MPK1/2/7 module through transcriptional regulation of MAPKKK14 (MAP3K14) (Sözen et al. 2020). In the transgenic line overexpressing *mSDE460*, many DEGs were assigned to Ethylene signaling group (Fig. 5). These investigations suggested that the regulation module of WRKY33 and ACS6 affected ethylene signaling in response to *Ca*LasSDE460 effector via the similar mechanism (Li et al., 2012). It has been shown that many genes involved in MAPK signaling pathway were highly upregulated in HLB-tolerant rough lemon (Yu et al. 2017). Thus, clarifying MAPKKK14 and WRKY33 functions in response to HLB should be an important point to understand the negative effect of *Ca*LasSDE460 effector on HLB-resistance of citrus in further research.

Heat shock proteins (HSP) are usually induced in the heat stress response, and the increase of temperature is accompanied by synthesis of heat shock proteins (Jiang et al. 2020). The synthesis of HSP70 under heat stress is involved in the regulation of photosynthetic carbon metabolism, which is proved to be necessary for the chloroplast development of *Arabidopsis thaliana* during heat stress (Zhong et al. 2013). Hsp70-Hsp90 multichaperone complex involved in the proper folding of many cytosolic and organelle proteins. it was shown that the folding of chloroplast proteins depends on the cooperative action of the chloroplast Hsp70-Hsp90 machineries (Willmund et al. 2008). The chloroplast HSP70 plays a crucial role in the maintenance and biogenesis of thylakoid membranes (Liu et al. 2007). Here, many HSP genes including HSP90 and HSP70 were affected by *mSDE460* expression and most of them were upregulated (Fig. 5). We noted that the expression of a HSP70b homology (*Cs_ont_1g010380*) was remarkably increased by *mSDE460* expression when temperature rose from 25 °C to 32 °C (Table 1 and Fig. 6). Meanwhile, the photosynthesis process (Carbon fixation in photosynthetic organisms, Porphyrin and chlorophyll metabolism, and Photosynthesis-antenna proteins)-related transcriptions were suppressed by expression of *mSDE460* at 32 °C. These data indicated high expression of the HSP70b homology maybe related to the change of photosynthesis process at 32 °C. Further, all the HSP DEGs were also assigned to the “protein processing in endoplasmic reticulum” pathway, which was positively affected by *mSDE460* expression (Fig. 4C). It has become clear that the endoplasmic reticulum (ER) plays an important role in immune signaling. For example, many pathogenesis-related proteins, R proteins, Plant receptors for Microbe-associated molecular patterns (MAMPs), and anti-microbial proteins secreted to the apoplast need to pass through the ER to execute their functions (Kørner et al., 2015), and it is also believed that high protein folding and secretion capacity of the ER is crucial for rapid and effective host immune responses (Wang et al. 2005). Totally, the above data suggested that HSPs have a crucial role in *Ca*LasSDE460 effector-regulated immunity response through ER processing in citrus.

It is broadly accepted that SA and JA signals act as master players in plant defense responses against numerous pathogens (Toruño et al. 2016). The presented study showed that *mSDE460* expression significantly affected plant hormone signal transduction pathway (Supplementary Fig. 2). *Cs_ont_1g027050* gene, which encodes salicylic acid (SA) carboxyl methyltransferase (*CsSAMT1*) involved in SA biosynthesis, was suppressed by *mSDE460* expression (Table 1). SAMT1 is responsible for the formation of MeSA from SA in plants (Zubieta et al. 2003). SA-mediated innate defenses play central roles in citrus response to HLB (Martinelli et al., 2013; Aritua et al., 2013; Zou et al., 2021). Our previous study confirmed that *CsSAMT1* gene promoted citrus resistance to HLB through activating the exchange of MeSA and SA signals (Zou et al., 2021), indicating *mSDE460* expression negatively affected CsSAMT1-mediated resistance to HLB. A LOX2 gene (*Cs_ont_2g033670*), encoding chloroplast lipoxygenase required for jasmonic acid accumulation (Bell et al. 1995), was also downregulated by *mSDE460* expression (Table 1). LOXs play a key role in defense responses against pathogen attack (Christensen et al. 2014). A 13-Lipoxygenase, GhLOX2, positively regulates cotton tolerance against *Verticillium dahliae*. LOX2 is a 13-LOX member, which belongs to non-heme, iron-containing oxidoreductases. 13-LOX catalyzes the oxidation of linolenic acid to generate 13-HPOT (hydroperoxy octadecatrienoic acid), which is metabolized to plant signaling compounds including JA (Maccarrone et al. 2001). Additionally, a NCED3 gene, which encodes 9-cis-epoxycarotenoid dioxygenase, a key enzyme in the biosynthesis of abscisic acid (ABA) (Zeevaart et al., 1988) was down-expressed at 25 °C, and but up-expressed at 32 °C (Table 1). It has been shown that ABA trigger citrus canker, a destructive bacterial disease induced by *Xanthomonas citri subsp. citri* (Long et al. 2019), indicating ABA signaling widely involved in regulation of disease resistance in citrus.

In conclusion, our study indicated that *Ca*LasSDE460 effector inhibited citrus immunity defense response through disturbing MAPK signaling, the “protein processing in endoplasmic reticulum” and the plant hormone signal transduction pathways. Several important genes in response to *Ca*LasSDE460 effector were identified. However, how the *Ca*LasSDE460 effector regulated these genes to alter citrus HLB-resistance remains to be verified, which merits further research. Additionally, determining the *Ca*LasSDE460 effector directly targeted specific host proteins or genes is also the key of further understanding the pathogenic mechanism of *Ca*Las in citrus.

## Materials and methods

### Plant and pathogen materials and growth conditions

Citrus materials containing *Ca*Las pathogen were obtained from Guangxi orchards. Reproduction and preservation of *Ca*Las was performed by grafting these citrus materials on healthy Wanjincheng orange (*C. sinensis* Osbeck) seedlings growing for two years in a greenhouse with restricted access.

Citrus materials for genetic transformation were obtained from the National Citrus Variety Improvement Center, Chongqing, China. All plant materials including transgenic plants were grown in a greenhouse maintained under 16 h photoperiod of 45 µmol m^−2^ s^−1^ illumination with 60% relative humidity at 28 °C.

### Construction of plant expression vector overexpressing *mSDE460*

Total RNA was isolated from the midrib tissues of Wanjincheng orange plants infected with *Ca*Las, and then was reverse transcribed into cDNA. Using the cDNA as template, *mSDE460* gene encoding mature secretory protein *Ca*LasSDE460 (CLIBASIA_00460) was amplified by T-SDE460-f/r primers (Supplementary Table 1), and PCR products were cloned into pGEM-Teasy (Promega, WI, USA). The PCR reaction system is 20µL, including 10µL high fidelity enzyme (TaKaRa, Ojin, Japan), 1µL forward, reverse primer (10 mM·L^−1^), 7µL ddH_2_O and 1µL cDNA (0.5 × 10^–5^ ng·L^−1^). PCR amplification conditions were as follows: pre-denaturation at 98 °C for 3 min, then conducted 35 amplification cycles (each at 98 °C for 30 s, 58 °C for 30 s and 72 °C for 30 s), and finally extend at 72 °C for 3 min. The sequence of *mSDE460* was determined by Sanger sequencing. Then, the *mSDE460* was unloaded from the pGEM-Teasy by *Bam*HI/*Sa*lI and inserted into a *Bam*HI/*Sa*lI-digested pLGN vector to construct the *p35S:mSDE460* vector. In this vector, the expression of *mSDE460* was driven by a 35S strong promoter (Fig. 1A). The vector was transformed into an EHA105 strain and transformants were confirmed by restriction enzyme and PCR analysis.

### Citrus transformation

*Agrobacterium tumefaciens-*mediated transformation of Wanjincheng oranges was conducted as described previously (Peng et al., 2015). Transgenic shoots were identified by GUS staining and PCR amplification, and was micro-grafted into Troyer citrange [*Poncirus trifoliata* (L.) Raf. × *C. sinensis* (L.) Osbeck] seedlings in vitro (Zou et al., 2019). After 4 weeks, transgenic plants were grafted onto the trifoliate orange rootstock in the greenhouse.

### Evaluation of HLB tolerance in transgenic plants

HLB tolerance in *mSDE460* transgenic plants was evaluated according to the method of Zou et al. (2017). Transgenic lines and wild type (WT) plants were firstly propagated by grafting on Troyer citrange rootstock in the greenhouse. After 1 year, 9 well-grown healthy plants per line were grafted with the Wanjincheng orange branches containing *Ca*Las. 3 grafted plants per line were cultured in greenhouse to evaluate HLB tolerance of transgenic plants. The *Ca*Las contents of the transgenic plants was detected and HLB symptoms were assessed at 3, 6, 9 and 12 months after infection. To investigate effects of temperature on the pathogenicity of *Ca*LasSDE460 effector, the rest of the grafted plants (3 plants per line) were inoculated with *Ca*Las and were grown at 25 °C (A) and 32 °C (B) for 3 months in constant-temperature incubator, and the *Ca*Las contents of the transgenic plants was detected and the HLB symptoms were assessed every month.

The populations of *Ca*Las bacteria (*Ca*Las cells ug^−1^ of citrus DNA) were determined by qPCR (Zou et al. 2017). 3 leaves per plant were randomly harvested to extract midrib tissues, and then mixed them together to extract genomic DNA. The number of *Ca*Las 16S rRNA genes and citrus 18S rRNA genes in the isolated DNA samples were detected using Cs16S-f/Cs16S-r and Cs18S-f/Cs28S-r primers (Supplementary Table 1), respectively. The qPCR reaction system is 12 µL, including 6 µL the SYBRPRIME qPCR Kit (Bioround Biotechnology, Chongqing, China), 0.3 µL forward and reverse real-time primer (10 mM·L^−1^), 4.4 µL ddH_2_O and 1 µL DNA (10 ng·µL^−1^). The PCR amplification conditions were: treatment at 95 °C for 2 min, then 40 amplification cycles (each at 65 °C for 10 s, 95 °C for 5 s), and finally extension at 60 °C for 15 s. Using citrus 18S gene as internal reference, the populations of *Ca*Las bacteria was determined according to the report of Zou et al. (2017). The HLB disease intensity of each transgenic line was estimated based on the bacterial populations in three plants.

### Construction of RNA-Seq libraries and high-throughput sequencing

The leaves of the healthy OE-5 transgenic line and healthy WT controls were sent to Biomarker technology Co., Ltd. (Beijing, China) for RNA-Seq. Three biological replicates were undergone in the test. The total RNA of citrus leaves was extracted using TRIzol reagent (Invitrogen company of the United States), and the total RNA was treated with DNase I reagent (Baoriyi Biotechnology Co., Ltd., Beijing, China). RNA concentration and purity were measured using NanoDrop 2000 (Thermo Fisher Scientific, Wilmington, DE). NanoDrop 2000 was used to measure RNA concentration and purity (Thermo Fisher Scientific, Wilmington, DE). RNA integrity was evaluated, and a sequencing library was established and then sequenced with the Illumina Hiseq 2500 platform (Biomarker technology Co., Ltd.).

### Analysis of RNA-seq data

All the clean reads were mapped to the reference genome of sweet oranges (http://citrus.hzau.edu.cn/orange/) using the HISAT 2.0.5 software (Kim et al. 2015). Gene function was annotated based on Nr, Nt, Pfam, KOG/COG, Swiss-Prot, KO and gene ontology (GO) databases. Gene expression levels in all the samples were estimated using the FPKM method (Mortazavi et al. 2008). Compared to WT control, differentially expressed genes (DEG) were identified using the DESeq2 package (Love et al. 2014). Genes with |log2 fold change|> 1 and adjusted *P*-values < 0.05 were defined as differentially expressed genes (DEGs). GO function and KEGG pathway enrichment analysis were performed using the GOseq R package (Young et al. 2010) and KOBAS software (Mao et al. 2005), respectively.

To analyze the pathways and functions regulated by *mSDE460* in detail, the DEGs were further annotated using MapMan software (Zhang et al. 2015). Differentially represented MapMan pathways and functions were defined using a two-tailed Wilcoxon rank sum test corrected using the Benjamin–Hochberg method (false discovery rate < 0.05).

### RT-qPCR analysis

With reference to the method in the kit manual, use the EASYspin plant RNA extraction kit to extract citrus RNA (Aidlab, Beijing, China). Use DS-11 spectrophotometer (DeNoVIX Inc., USA) to measure the absorbance ratio of the sample at 260 nm and 280 nm to determine the RNA concentration and purity.

Citrus total RNA was isolated using the EASYspin plant RNA extraction kit following the manufacturer’s instructions (Aidlab, Beijing, China). RNA was reverse transcribed into cDNA using the Prime ScriptRT Master Mix (TaKaRa, Ojin, Japan). RT-qPCR reaction system is 12 µL, including 6 µL the SYBRPRIME qPCR Kit (Bioround Biotechnology, Chongqing, China), 0.3 µL forward and reverse real-time primer (10 mM·L^−1^), 4.4 µL ddH_2_O and 1 µL cDNA (10 ng·µL^−1^). The PCR amplification conditions were: treatment at 95 °C for 2 min, then 40 amplification cycles (each at 65 °C for 10 s, 95 °C for 5 s), and finally extension at 60 °C for 15 s. Using citrus GAPDH gene (Mafra et al., 2012) as internal reference gene, the relative expression of *mSDE460* in transgenic plants was calculated by the 2^−ΔΔCt^ method (Livak and Schmittgen, 2001). The primers were listed in Supplementary Table 1. The test was repeated three times.

## Statistical analyses

Statistical analyses of all data were conducted in Excel, using the Student’s *t*-test to compare differences between the control and samples at 5% significance level.

### Supplementary Information

**Additional file 1:** **Supplementary Table 1.** The primers used in the study. **Supplementary Table 2.** Summary of sequencing data for each sample. **Supplementary Table 3.** Mapping summary of sequencing data for each sample. **Supplementary Table 4.** Differentially expressed genes in the OE-5 line compared to WT control at 25 °C. **Supplementary Table 5.** Differentially expressed genes in the OE-5 line compared to WT control at 32 °C. **Supplementary Table 6.** KEGG enrichment analysis of differentially expressed genes in the OE-5 line. **Supplementary Table 7.** Differentially expressed genes in WT at 32 °C compared to 25 °C. **Supplementary Table 8.** Differentially expressed genes in OE-5 at 32 °C compared to 25 °C. **Supplementary Table 9.** KEGG enrichment analysis of differentially expressed genes in WT at 32 °C compared to 25 °C. KEGG enrichment analysis of differentially expressed genes in OE-5 at 32 °C compared to 25 °C.

**Additional file 2:** **Figure S1.** Repeated correlation assessment Pearson's Correlation Coefficient (r). **Figure S2.** Enrichment analysis of differentially expressed gene KEGG pathway at 25 °C and 32 °C. **Figure S3.** Enrichment analysis of differentially expressed gene KEGG pathway in WT (A) and OE-5 transgenic (B) plant at 32 °C compared to 25 °C. **Figure S4.** MapMan visualizes the functional categories of genes differentially expressed in WT (A) and OE-5 (B) transgenic citrus at 32 °C using that at 25 °C as control. **Figure S5.** The differentially expressed genes in OE-5 transgenic plant at 25 °C and 32 °C were verified by using qPCR. **Figure S6.** The differentially expressed genes in wildtype (WT) and OE-5 transgenic plants at 32 °C compared to 25 °C were verified by using qPCR.

## Data Availability

The RNAseq data (FASTQ files) underlying this article will be shared upon request to the corresponding author.

## Abbreviations

DEGs: Differentially Expressed Genes
SRA: Sequence Read Archive
HLB: Huanglongbing
*Ca*Las: *Candidatus* Liberibacter asiaticus
mSDE460: Mature secretion protein of *Ca*LasSDE460
*Ca*Laf: *Candidatus* Liberibacter africanus
*Ca*Lam: *Candidatus* Liberibacter americanus
SDEs: Sec-dependent effectors
*E. coli*: *Escherichia coli*
PhoA: Phosphatase
SAR: Systemic acquired resistance
GUS: Glucuronidase
WT: Wild type
Nr: NCBI non-redundant protein sequences
Nt: NCBI non-redundant nucleotide sequences
Pfam: Protein family
COG: Clusters of Orthologous Groups of proteins
Swiss-Prot: Swiss-Prot Protein Sequence Database
KO: KEGG Ortholog database
COGs: Clusters of Orthologous Groups
GO: Gene Ontology
FPKM: Fragments Per Kilobase of Exon Per Million Fragments Mapped
KEGG: Kyoto Encyclopedia of Genes and GenomesGene Ontology
GAPDH: Glyceraldehyde-3-phosphate dehydrogenase
OE: Overexpression
MAPK: Mitogen-activated Protein Kinase
ABC: ATP binding cassette
PR: Pathogenesis-related
SA: Salicylic acid
JA: Jasmonic acid
MAPKKK14: Mitogen-activated protein kinase kinase kinase 14
RT-qPCR: Real Time Quantitative PCR
bHLH: Basic helix-loop-helix
*N. Benthamiana*: *Nicotiana benthamiana*
HSP: Heat shock protein
ER: Endoplasmic reticulum
MAMPs: Microbe-associated molecular patterns
SAMT1: Salicylic acid carboxyl methyltransferase
MeSA: Methyl salicylate
ABA: Abscisic acid
Erd9: Early-responsive to dehydration 9
CML37: Calmodulin like 37
HSC: Heat shock cognate
GSTL3: Glutathione transferase L3
