# Supplementary material for: Function and molecular mechanism analysis of CaLasSDE460 effector involved in the pathogenesis of “Candidatus Liberibacter asiaticus” in citrus
Source: Mol Hortic. 2023 Jul 24;3:14. doi: 10.1186/s43897-023-00062-3 (PMC10514941; doi:10.1186/s43897-023-00062-3)
Supplement: Supplementary file 2 — Additional file 2: Figure S1. Repeated correlation assessment Pearson's Correlation Coefficient (r). Figure S2. Enrichment analysis of differentially expressed gene KEGG pathway at 25 °C and 32 °C. Figure S3. Enrichment analysis of differentially expressed gene KEGG pathway in WT (A) and OE-5 transgenic (B) plant at 32 °C compared to 25 °C. Figure S4. MapMan visualizes the functional categories of genes differentially expressed in WT (A) and OE-5 (B) transgenic citrus at 32 °C using that at 25 °C as control. Figure S5. The differentially expressed genes in OE-5 transgenic plant at 25 °C and 32 °C were verified by using qPCR. Figure S6. The differentially expressed genes in wildtype (WT) and OE-5 transgenic plants at 32 °C compared to 25 °C were verified by using qPCR. [file 43897_2023_62_MOESM2_ESM.docx]

**Function and molecular mechanism analysis of *Ca*LasSDE460 effector involved in the pathogenesis of** **“*Candidatus* Liberibacter asiaticus” in citrus**

Shuai Wang^a,1^, Meixia Du^a,1^, Liting Dong^a,1^, Rongrong Qu^a^, Danlu Ran^a^, Juanjuan Ma^a^, Xuefeng Wang^a^, Lanzhen Xu^a^, Weimin Li^b^, Yongrui He^a^, Xiuping Zou*

^a^ Citrus Research Institute, Southwest University/National Citrus Engineering Research Center, Chongqing, People’s Republic of China

^b^ Key Laboratory for Northern Urban Agriculture of Ministry of Agriculture and Rural Affairs, Beijing University of Agriculture, Beijing, People’s Republic of China

* Corresponding authors. E-mail addresses: [zouxiuping@cric.cn](mailto:zouxiuping@cric.cn)

^1^ These authors contributed equally to this work.


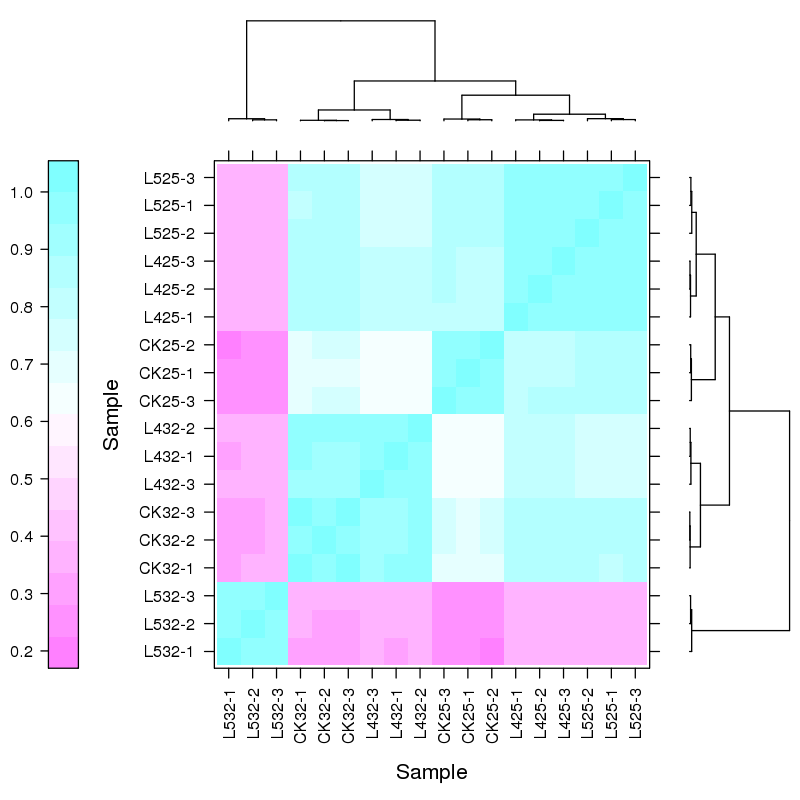


**Supplementary Figure 1. Repeated correlation assessment Pearson's Correlation Coefficient (r)**

（A）

25℃


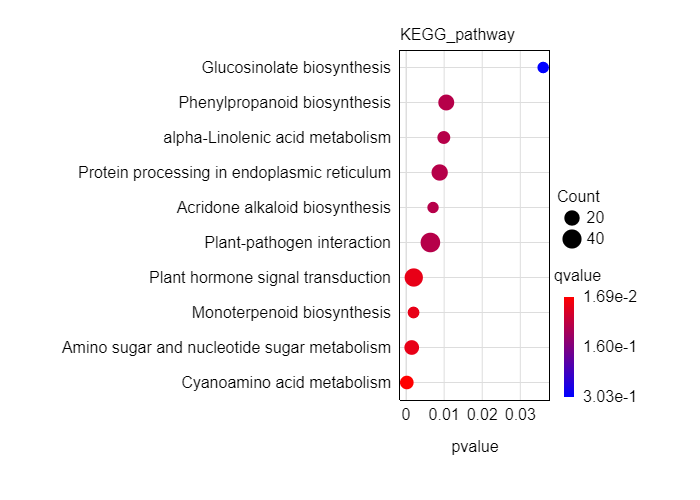


（B）

32℃


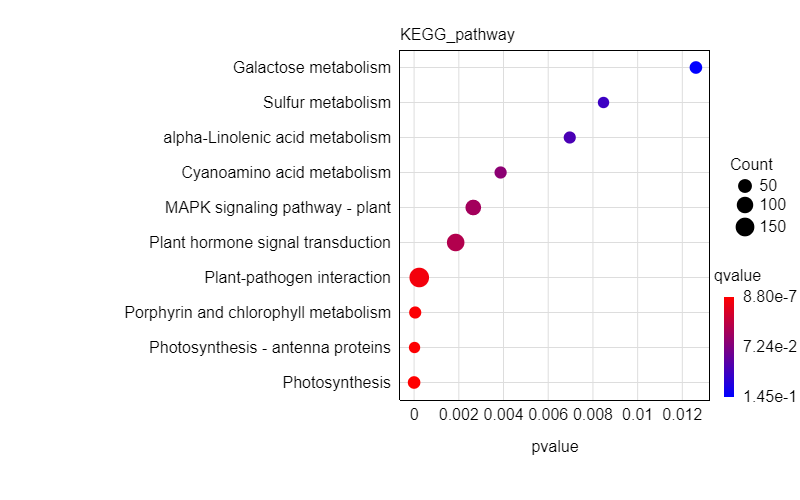


**Supplementary Figure 2. Enrichment analysis of differentially expressed genes KEGG pathway at 25 ℃ and 32 ℃.** The figure shows the top 10 pathways (qvalue <0.05, Fisher’s exact test). Each circle represents a KEGG pathway, the ordinate represents the pathway name, the abscissa represents the enrichment factor, the color of the circle represents qvalue, and the size of the circle represents the number of genes enriched in the pathway. **(A)** At 25 ℃, the differential genes were mainly significantly enriched in the Cyanoamino pathway. **(B)** At 32 ℃, the differential genes were mainly significantly enriched in the Photosynthesis pathway.


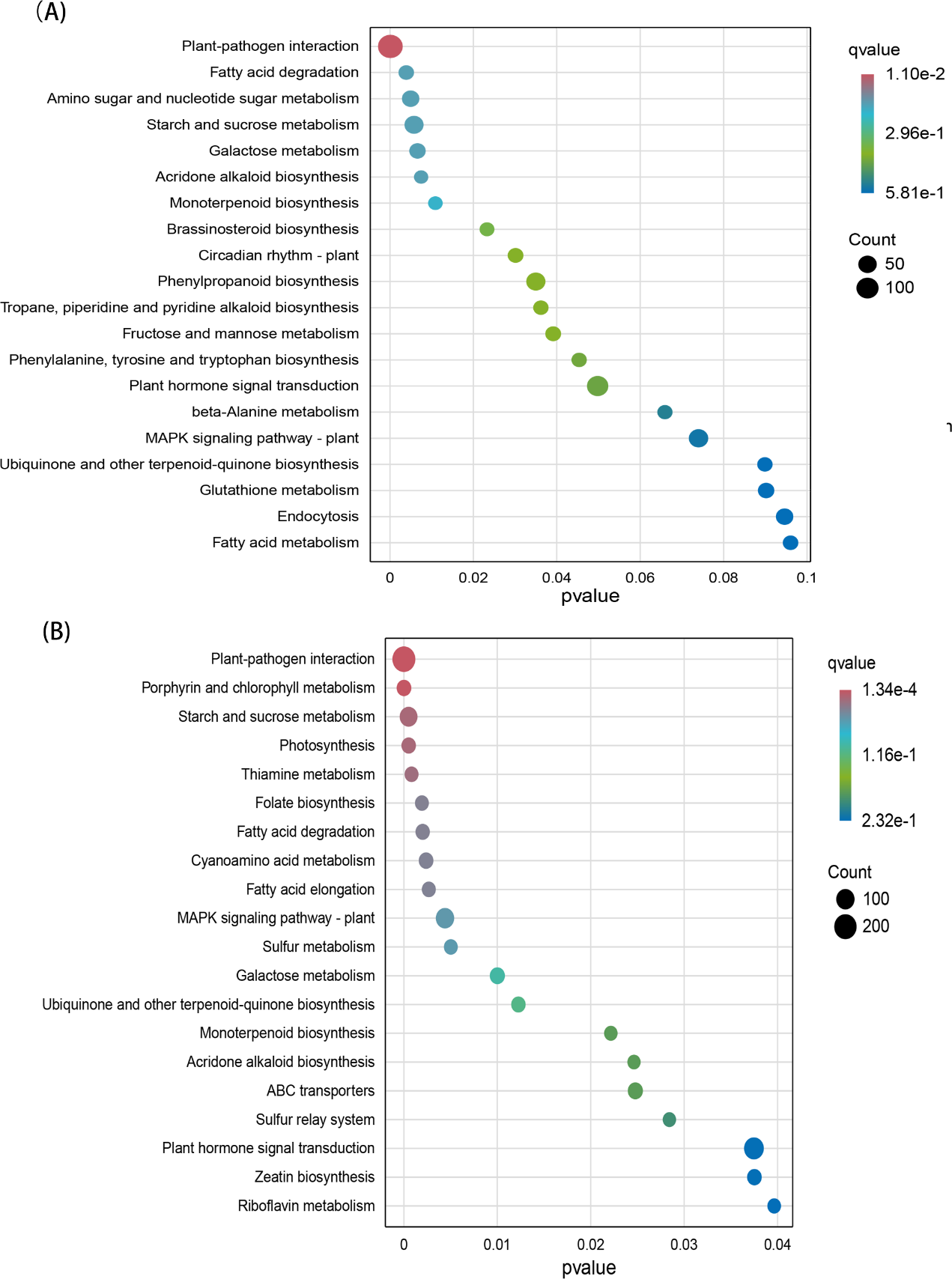


**Supplementary Figure 3.** **Enrichment analysis of differentially expressed gene KEGG pathway in WT and OE-5 transgenic plant at 32 ℃ compared to 25 ℃.** The figure shows the top 20 pathways (qvalue <0.05, Fisher’s exact test). Each circle represents a KEGG pathway, the ordinate represents the pathway name, the abscissa represents the enrichment factor, the color of the circle represents qvalue, and the size of the circle represents the number of genes enriched in the pathway. **(A)** In WT plant, the differential genes were mainly significantly enriched in the Plant-pathogen interaction pathway. **(B)** In OE-5 transgenic plant, the differential genes were mainly significantly enriched in the Plant-pathogen interaction pathway.

**
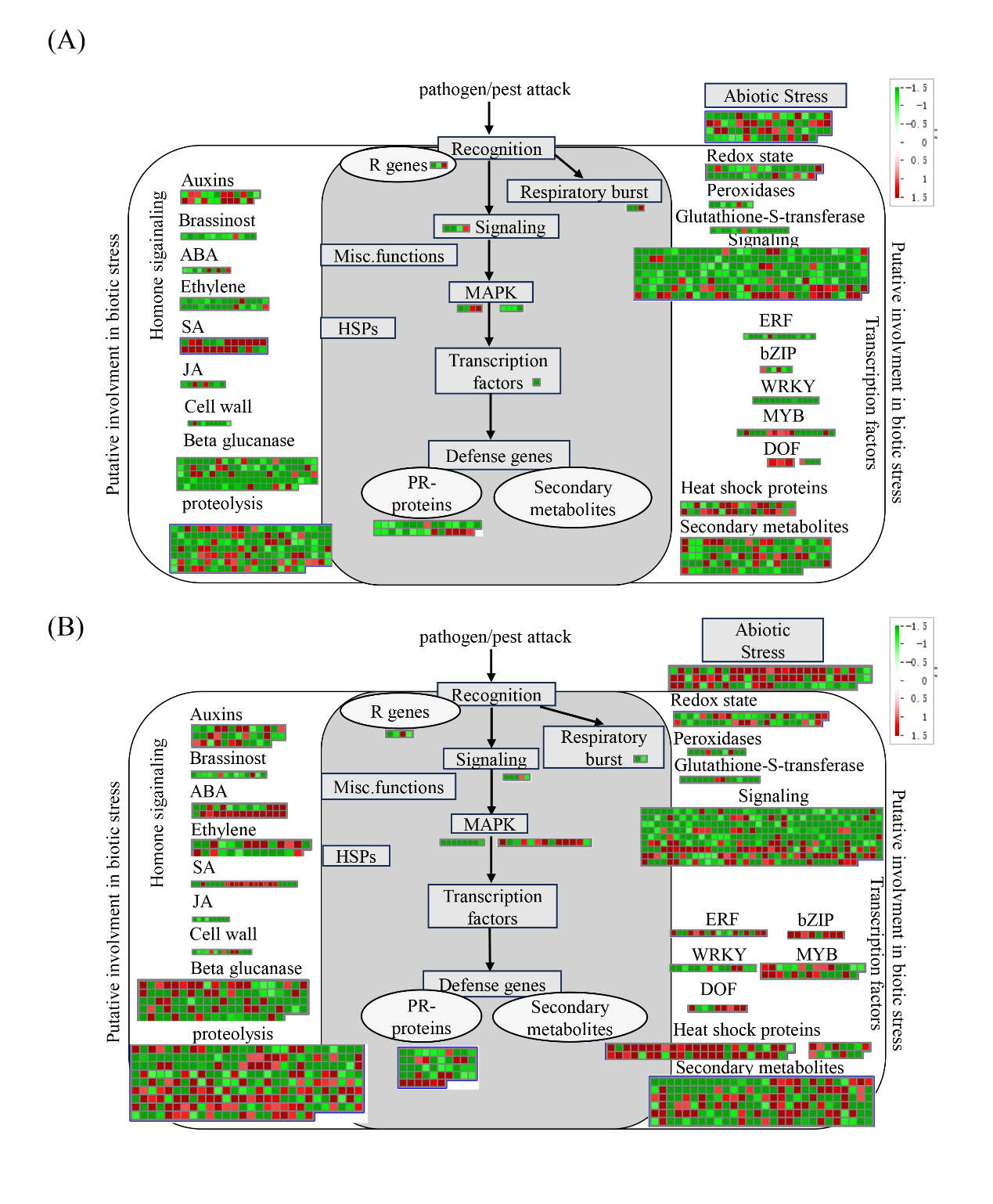
**

**Supplementary Figure 4.** **MapMan visualizes the functional categories of genes differentially expressed in WT (A) and OE-5 (B) transgenic citrus at 32 ℃ using that at 25 ℃ as control.** Every square block indicates a DEG. Significantly up-regulated and down-regulated genes are displayed in red and green, respectively.


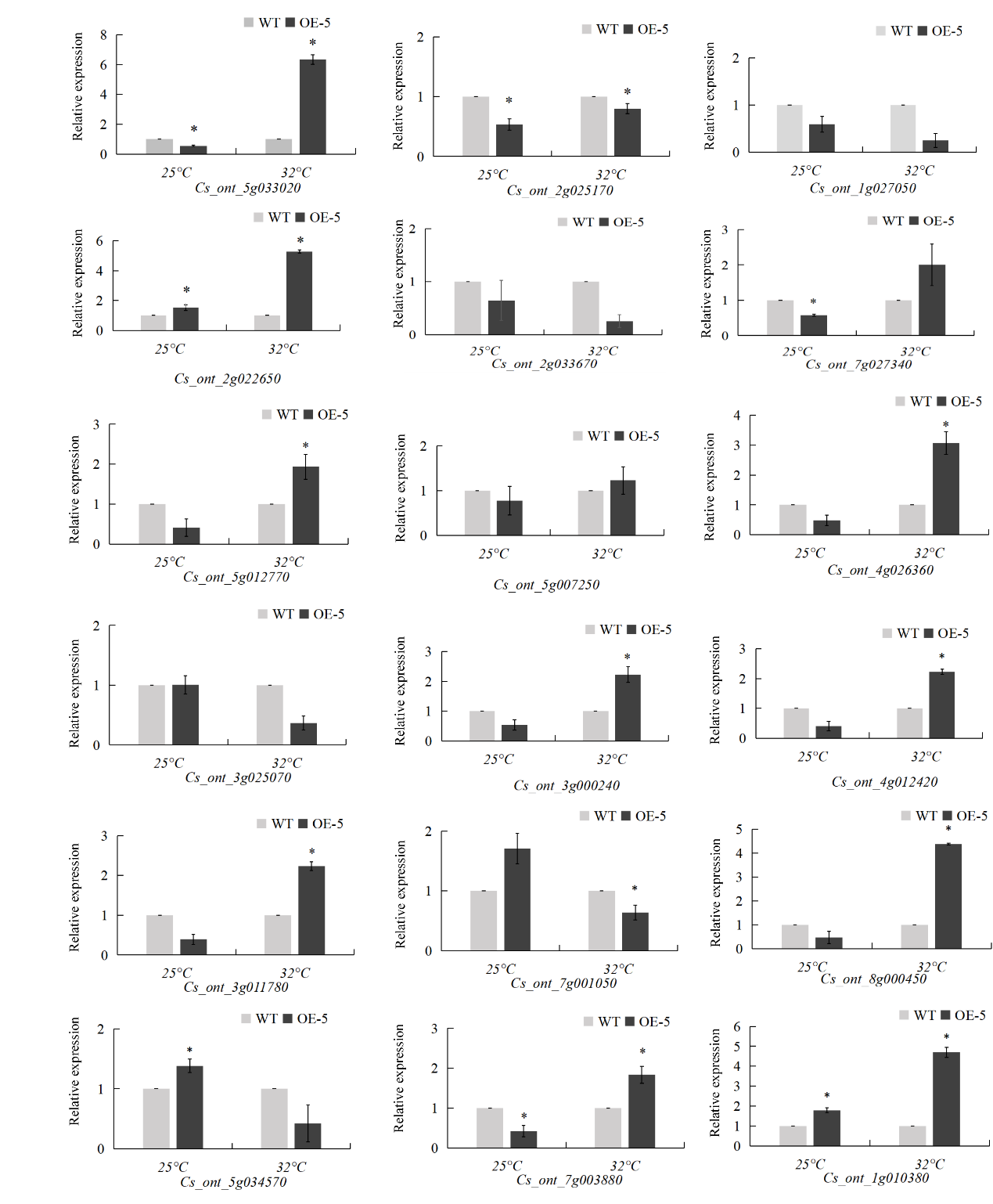


**Supplementary Figure 5.** **The differentially expressed genes in OE-5 transgenic plant at 25 ℃ and 32 ℃ were verified by using qPCR.**

**
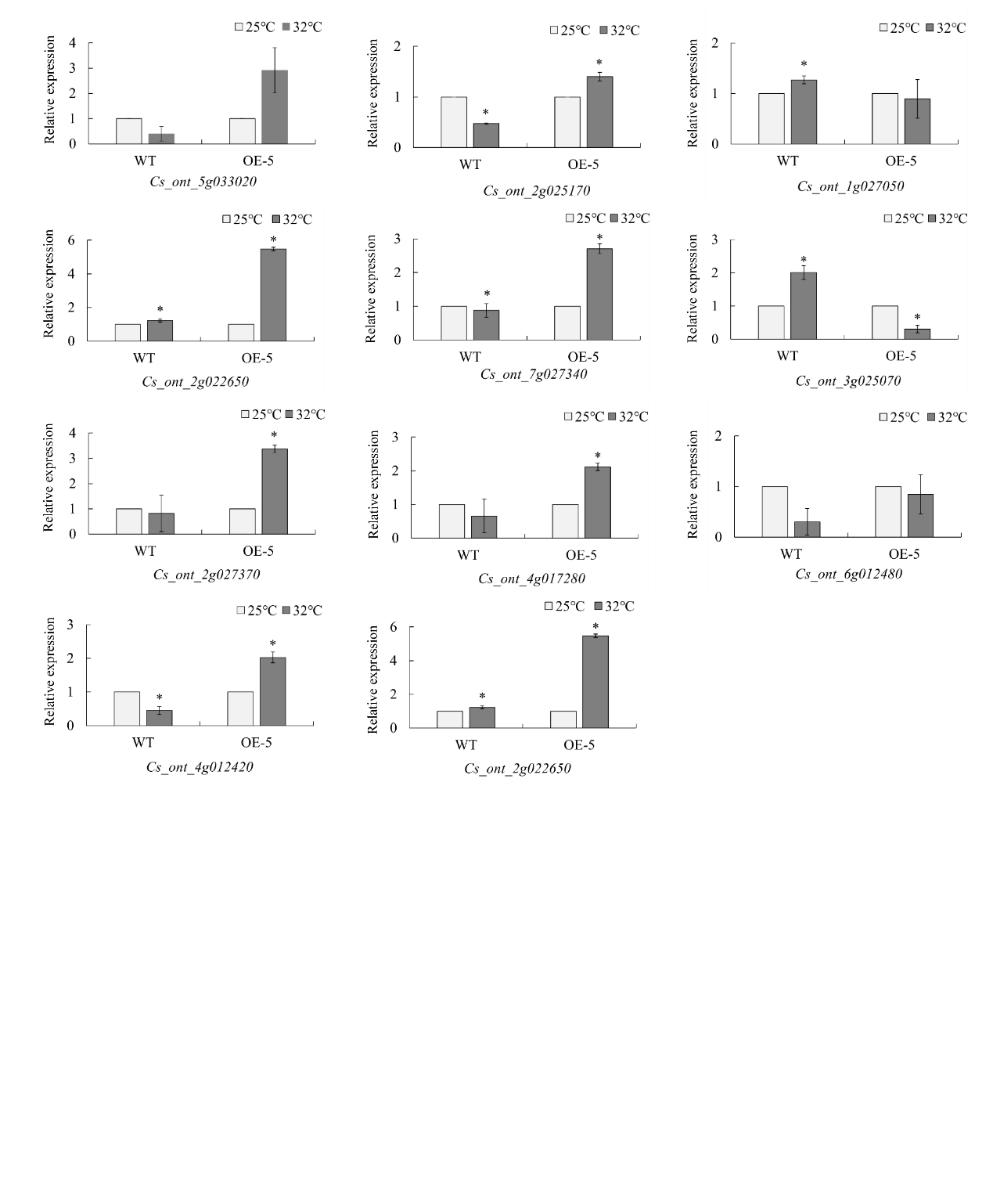
**

**Supplementary Figure 6.** **The differentially expressed genes in wildtype (WT) and OE-5 transgenic plants at 32 ℃ compared to 25 ℃ were verified by using qPCR.**
